# First report of sporotrichosis by *Sporothrix brunneoviolacea*

**DOI:** 10.1016/j.mmcr.2025.100756

**Published:** 2025-11-27

**Authors:** Mariana Rodrgiues Trápaga, Bram Spruijtenburg, Bruna Jacomel, Jéssica Estefânia Dávila Hidalgo, Karine Ortiz Sanchotene, Fabiana Fedatto Bernardon, Bruna Muradás Esperon, Vanice Rodrigues Poester, Theun de Groot, Eelco F.J. Meijer, Melissa Orzechowski Xavier

**Affiliations:** a Programa de Pós-graduação em Ciências da Saúde, Faculdade de Medicina (FaMed), Universidade Federal do Rio Grande (FURG), Rio Grande, RS, Brazil; b Laboratório de Micologia, Faculdade de Medicina (FaMed), Universidade Federal do Rio Grande (FURG), Rio Grande, RS, Brazil; c Department of Medical Microbiology, Radboudumc, Nijmegen, the Netherlands; d Radboudumc-CWZ Center of Expertise for Mycology, Nijmegen, the Netherlands; e Department of Medical Microbiology and Immunology, Canisius-Wilhelmina Hospital (CWZ)/Dicoon, Nijmegen, the Netherlands; f Postgraduate Program in Microbiology, Parasitology and Pathology, Biological Sciences, Department of Basic Pathology, Federal University of Parana, Curitiba, Brazil

**Keywords:** Sporotrichosis, Zoonotic transmission, Feline, Molecular identification, *Sporothrix*

## Abstract

Sporotrichosis is a neglected fungal disease, affecting mammals. Here, a feline patient presented a small ulcerated lesion. Short treatment by itraconazole resulted in clinical cure. The isolate was identified as *Sporothrix brunneoviolacea* by sequencing. Despite proper growth, antifungal susceptibility testing by microbroth dilution could not be performed due to a lack of growth. This is the first report of sporotrichosis by *S. brunneoviolacea*, which was originally classified as an environmental and non-pathogenic species.

## 1 Introduction

Sporotrichosis is a neglected fungal disease reported on all inhabited continents [1]. Originally classified as an implantation mycosis, inhalation of fungal spores or mucosal contact with lesions of infected hosts are also recognized as transmission routes [1]. Humans and domestic cats (*Felis catus*) are most affected by this disease, although infections in other mammals such as dogs (*Canis familiaris*) and rodents are occasionally reported [2]. Sporotrichosis is most severe in cats and is often fatal when not treated with proper antifungal treatment and diagnosed in an early stage of the disease [3]. For humans and other mammals, fixed cutaneous is the most common presentation, followed by lymphocutaneous, disseminated, and ocular forms [2,4,5].

Although over 50 species of the *Sporothrix* genus are described, infections are mainly restricted to species of the pathogenic clade that comprises *S. schenckii*, *S. globosa*, *S. brasiliensis* and *S. luriei* [6]. Other *Sporothrix* species are usually classified as environmental and are rarely implicated in disease [6]. In South America, *S. brasiliensis* infections are highly prevalent and cases are mainly driven by colonized or infected cats [7]. Conversely, *S. schenckii* and *S. globosa* are the most common agents in Europe and Asia and are often caused by sapronotic transmission [2]. Interestingly, zoonotic transmission of *S. schenckii* by cats has also been reported in Asia, although other *Sporothrix* species are not implicated in this transmission type so far [8]. Regardless of the host, itraconazole is the drug of choice, with terbinafine as an alternative therapeutic option [4,9]. Worryingly, itraconazole resistant *S. brasiliensis* and *S. schenckii* isolates have been reported recently, although a correlation with clinical outcomes remains to be investigated [10]. Here, we report the first case of sporotrichosis by *Sporothrix brunneoviolacea* in a feline patient in Brazil, which was successfully treated with itraconazole.

## 2 Case presentation

**Fig. 1:**
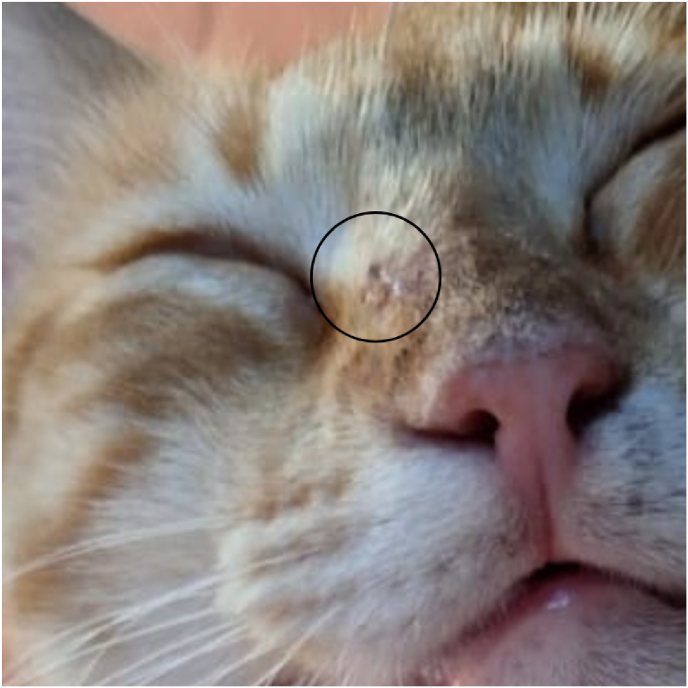
Cutaneous lesion in regressive process after 30 days of itraconazole in the cat infected by *Sporothrix brunneoviolacea*.

On January of 2024, a 8-months-old, male, non-neutered and antifungal naive cat of not defined breed with access to the street and without known underlying disease was presented to a veterinary clinic in Rio Grande, Rio Grande do Sul, Brazil. A discrete edema with small ulcerated and secretive lesions were present on the nose (Fig. 1). The veterinarian sent a swab sample in Stuart transport medium for diagnosis to the Mycology Laboratory (MycoLab) of the Faculty of Medicine from Federal University of Rio Grande. The swab was cultured on plates with Sabouraud dextrose agar with and without cycloheximide (Kasvi ®, São José dos Pinhais, PR, Brazil) at 28 °C for 7 days.

After 7 days a positive culture was obtained, which was identified as *Sporothrix* sp. by macro and micromorphology. Antifungal treatment by itraconazole (100mg/day; 5mg/kg) capsules mixed with food was initiated for 60 days, resulting in clinical cure. The cat's owner also reported being scratched on the right hand by the cat, and subsequently, developed a small ulcerated lesion with lymphadenopathy. Erroneously she did not consult a clinician and reported cure by self-administration of itraconazole. No culture was obtained from the human patient.

For species identification DNA was extracted with the MagNA Pure and Viral NA Small Volume kit, Pathogen 200SV protocol and the MagNA Pure 96 instrument (all Roche Diagnostics GmbH, Mannheim, Germany) according to manufacturers’ instructions. Genomic libraries were prepared and sequenced with the Illumina Novaseq 6000 platform (Illumina, San Diego, CA, USA) with 2- by 150-bp paired-end-read mode. De novo assembly was performed with SPAdes v4.2.0 using default parameters. The calmodulin gene was identified by performing BLASTn search in the assembled genome using CaM sequence from *S. schenckii* (AM490340.1) as a reference. The resulting sequence was aligned with NCBI Nucleotide BLAST (https://blast.ncbi.nlm.nih.gov/Blast.cgi) indicating the species as *S. brunneoviolacea*. Next, reads were aligned against the *S. brunneoviolacea* reference CBS 124561 genome (GCA_021396205.1) using BWA-MEM v0.7.18, yielding a genomic coverage >99 %, confirming accurate species identification.

**Fig. 2:**
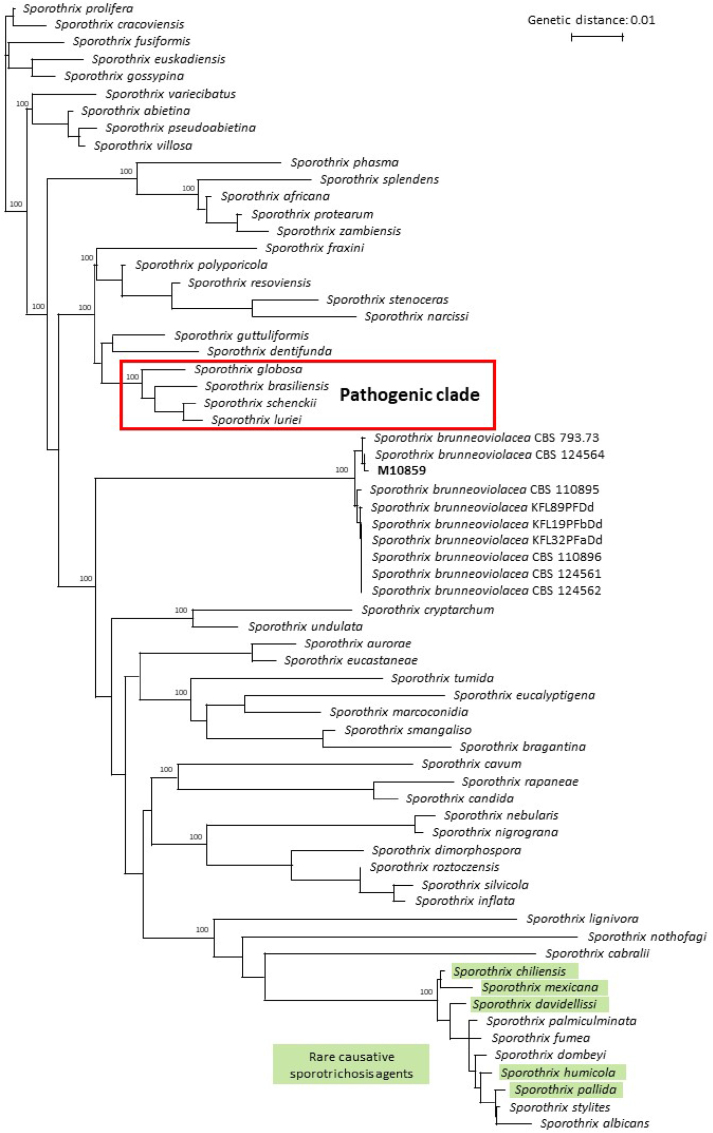
**Phylogenetic tree based on calmodulin sequences of *Sporothrix* species, including the current isolate** M10859 **indicated in bold.** Bootstrap values of 100 are shown above the nodes.

The resulting *CaM* sequence was compared to all available *Sporothrix* species and additional *Sporothrix brunneoviolacea* isolates present in the National Center for Biotechnology Information Nucleotide database (Table S1). Nucleotide alignment was done using MAFFTv7 and the phylogenetic tree was built with IQ-TREE web server as described earlier [11]. Raw data generated in the current study was deposited to the NCBI Sequence Read Archive (SRA) database under BioProject ID PRJNA1252419. The current isolate differed with three nucleotide mismatches to the most closely related *S. brunneoviolacea* (CBS 124564) (Fig. 2). Interestingly, the species is only distant related to the clinical clade or other environmental species implicated with disease.

**Fig. 3:**
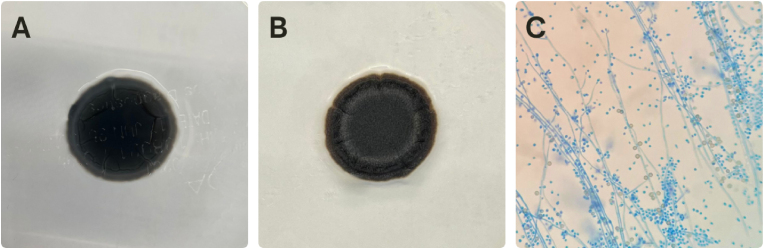
*Sporothrix brunneoviolacea* colony after 14 days of growth on Potato Dextroxe Ágar **(A)** Colony reverse; **(B)** Colony surface morphology; **(C)** Micromorphology showing lateral conidia stained with lactophenol cotton blue (x400).

Then, the colony characteristics were revised by subculture and slide culture. The colony was flat and velvety, with zonate coloration and grey tones. Microscopic identification revealed the presence of subhyaline, septate hyphae, conidiophores with terminal conidia and small conidia that were globose to oval. Additionally, lateral melanized conidia, a characteristic of *S. brunneoviolacea*, were observed, while diffusible violet-brown pigment, another characteristic of this species [12], was not produced (Fig. 3).

Next, isolates were grown for seven days on PDA (Difco Laboratories, Detroit, MI, USA) and antifungal susceptibility testing (AFST) was conducted at 30 °C with 72 hours of incubation with antifungals itraconazole (Janssen Cilag, Breda, The Netherlands), posaconazole (Merck), amphotericin B (Bristol Myers Squib, Woerden, The Netherlands), following microbroth dilution according to the Clinical & Laboratory Standards Institute (CLSI) reference method M38 and according to the European Committee on Antimicrobial Susceptibility Testing (EUCAST) E.def 9.4 [13,14]. Quality control strain *Aspergillus flavus* ATCC 204304 was included during the assays. While the quality control strain yielded minimum inhibitory concentrations (MICs) within the outlined ranges, *S. brunneoviolacea* did not grow at all when incubated according to CLSI and EUCAST microbroth dilution guidelines. Of note, inoculum was repeatedly incubated on different days and incubated for a total of 72 hours, with also the control well without antifungals present showing no growth.

## 3 Discussion

The current study identified for the first time a case of sporotrichosis caused by *S. brunneoviolacea*, found in a feline patient from Brazil. Moreover, this is the first description of *S. brunneoviolacea* in Brazil. In the last few decades, an increasing number of rare and novel fungal species have been reported in both animals and humans [15,16]. This is in part due to the increasing burden of fungal infections but also due to the adaptation of fungi to mammal hosts and advancements in species identification [17].

*Sporothrix brunneoviolacea* exhibits discrete distinct morphological characteristics compared to classical *Sporothrix* species of the pathogenic clade [12,15]. Its hyphae are often sub-hyaline, whereas species like *S. schenckii*, *S. brasiliensis* and *S. globosa* are typically fully hyaline. Another notable feature is the abundance of melanized lateral conidia, giving *S. brunneoviolacea* colonies a darker appearance. Texture of the colony typically shows a velvety surface, in contrast to the coriaceous aspect observed in the classical species. This species also grows slower at 30 °C than the other clinical species, and shows a limited growth at 37 °C, reinforcing its weak thermotolerance, and consequently minor virulence in mammalian hosts. The *S. brunneoviolacea* isolate in this report also exhibited all these characteristics. In contrast, the violet halo, a characteristic pigmentation for this species [12], was not clearly observed in our isolate, even after incubation at 30 °C, suggesting intraspecies variability although the influence of differences in culture conditions cannot be excluded.

With *CaM* sequencing, the isolate was identified as *S. brunneoviolacea*, while phylogenetic analysis demonstrated that this species formed a distinct branch within the genus. Earlier investigations found *S. brunneoviolacea* solely in soil from the USA and Poland [12]. The cat likely acquired the fungus from the environment by traumatic inoculation, as is common for most *Sporothrix* species [3]. Given that the owner reported clinical signs of sporotrichosis after being scratched, *S. brunneoviolacea* appears to be capable of zoonotic transmission, which is to date only reported for *S. schenckii* and *S. brasiliensis* [18]. However, it is important to highlight that the potential of zoonotic transmission of this species must be confirmed in future studies since the clinical sporotrichosis signs of the owner were self-reported. Furthermore, the owner lives in a hyperendemic area of cat-transmitted sporotrichosis by *S. brasiliensis*, being previously made aware of the zoonotic transmission of this fungi by local healthcare professionals.

Interestingly, sporotrichosis is predominantly caused by *S. schenckii*, *S. brasiliensis* and *S. globosa*, which are considered highly virulent [1]. Sporotrichosis cases in cats are often disseminated and antifungal treatment takes a prolonged time of usually 2 months or longer in felines [9]. Conversely, there are sporadic cases by species outside the pathogenic clade (e.g. *Sporothrix chilensis*, *Sporothrix mexicana* and *Sporothrix davidellisii*), which appear to display a lower level of virulence [19]. These cases are overall mild in presentation and antifungal treatment is usually effective within a short period [20]. The feline patient in the current study also displayed a mild form of the disease and was successfully treated with a short course of itraconazole, which is in accordance with earlier reports of ‘environmental’ *Sporothrix* species.

For *Sporothrix* species, treatment failure is reported although it remains unclear to which extent antifungal resistance is involved [10]. In order to trace and detect elevated MICs, surveillance with reference methods like CLSI or EUCAST microdilution are warranted. Surprisingly, both methods did not work for *S. brunneoviolacea* despite proper sporulation, while these have been extensively applied to species of the pathogenic clade [20]. To enable sufficient growth in microbroth dilution assays, a more nutrient-rich medium could be worthwhile to investigate. Nonetheless, itraconazole treatment resolved the lesions in both the cat and owner, suggesting antifungal activity.

The main limitation of this study is its retrospective nature that refrained us from collecting isolates from the owner and inspecting tissues to confirm the presence of *Sporothrix* in her lesion. Since no species identification and genotyping could be performed on the human case, zoonotic transmission cannot be confirmed [18].

To conclude, we report the first case of sporotrichosis by *S. brunneoviolacea* that was previously considered an environmental species with no pathogenic potential in mammals. The infected patient was a feline from southern Brazil with a small lesion, which likely infected the owner as well. A short course of itraconazole was sufficient to resolve the lesion. Using the *CaM* sequence, *S. brunneoviolacea* appeared not closely related to other pathogenic *Sporothrix* species. Surprisingly, microbroth dilution by CLSI and EUCAST guidelines did not yield any growth.

## CRediT authorship contribution statement

**Mariana Rodrgiues Trápaga:** Writing – review & editing, Methodology, Investigation, Formal analysis, Data curation, Conceptualization. **Bram Spruijtenburg:** Writing – original draft, Methodology, Investigation, Formal analysis, Data curation, Conceptualization. **Bruna Jacomel:** Investigation, Methodology, Writing – review & editing. **Jéssica Estefânia Dávila Hidalgo:** Investigation, Writing – review & editing. **Karine Ortiz Sanchotene:** Investigation, Writing – review & editing. **Fabiana Fedatto Bernardon:** Investigation, Writing – review & editing. **Bruna Muradás Esperon:** Investigation, Writing – review & editing.**Vanice Rodrigues Poester:** Writing – review & editing. **Theun de Groot:** Supervision, Writing – review & editing, Methodology, Investigation, Formal analysis. **Eelco F.J. Meijer:** Writing – review & editing, Supervision, Funding acquisition. **Melissa Orzechowski Xavier:** Writing – review & editing, Supervision, Funding acquisition.

## Ethical Form

Clinical data were retrieved from the LabMyco database and through a telephone interview with the owner, who assigned a consent form to answer some questions regarding the case. The study adheres to the Declaration of Helsinki, and was approved by the ethics committee of the Federal University of Rio Grande (99284218.5.0000.5324, 2022 and 64230922.8.0000.5324, 2024).

The statements on funding, conflict of interest and consent need to be submitted via our Ethical Form that can be downloaded from the submission site www.ees.elsevier.com/mmcr. **Please note that your manuscript will not be considered for publication until the signed Ethical Form has been received.**

## Conflict of interest

EFJM received research grants from Mundipharma and Scynexis, is in the scientific advisory board for Pfizer and has received speaker fees from Gilead Sciences. The other authors declare that they have no competing financial or personal interests which can influence the work reported in this paper.
