# Supplementary material for: First report of sporotrichosis by Sporothrix brunneoviolacea
Source: Med Mycol Case Rep. 2025 Nov 27;50:100756. doi: 10.1016/j.mmcr.2025.100756 (PMC12718152; doi:10.1016/j.mmcr.2025.100756)
Supplement: Multimedia component 1 [file mmc1.docx]

**Supplementary**

**Table S1: Calmodulin control sequences of 65 *Sporothrix* species included in the phylogenetic analysis.**

| **Species** | **ID** | **Accession number** |
| --- | --- | --- |
| *Sporothrix abietina* | CMW40454 | MW579752.1 |
| *Sporothrix africana* | 8.3 | MG431430.1 |
| *Sporothrix albicans* | CBS 302.73 | AM398396.1 |
| *Sporothrix aurorae* | CBS 118837 | KX590783.1 |
| *Sporothrix bragantina* | CBS 474.91 | KX590784.1 |
| *Sporothrix brasiliensis* | CBS 120339 | KP101421.1 |
| *Sporothrix brunneoviolacea* | CBS 793.73 | KP017106.1 |
| *Sporothrix brunneoviolacea* | CBS 110895 | KP017104.1 |
| *Sporothrix brunneoviolacea* | CBS 110896 | KP017102.1 |
| *Sporothrix brunneoviolacea* | CBS 124561 | KF574472.1 |
| *Sporothrix brunneoviolacea* | CBS 124562 | KX590803.1 |
| *Sporothrix brunneoviolacea* | CBS 124564 | KP017105.1 |
| *Sporothrix brunneoviolacea* | KFL19PFbDd | OP589043.1 |
| *Sporothrix brunneoviolacea* | KFL32PFaDd | OP589037.1 |
| *Sporothrix brunneoviolacea* | KFL89PFDd | OP589043.1 |
| *Sporothrix cabralii* | CMW 38098 | KX590804.1 |
| *Sporothrix candida* | CBS 129717 | KX590785.1 |
| *Sporothrix cavum* | KFL42215aDRJ | MW768976.1 |
| *Sporothrix chilensis* | Ss469 | KP711815.1 |
| *Sporothrix cracoviensis* | KFL2514bRJTD | MH283529.1 |
| *Sporothrix cryptarchum* | KFL410DB16bRJCU | MH741231.1 |
| *Sporothrix davidellissi* | CBS 147636 | PV092593 |
| *Sporothrix dentifunda* | CBS 115790 | KX590787.1 |
| *Sporothrix dimorphospora* | CBS 125442 | KX590806.1 |
| *Sporothrix dombeyi* | CBS 455.83 | KX590793.1 |
| *Sporothrix eucalyptigena* | CBS 139899 | KX273431.1 |
| *Sporothrix eucastaneae* | CBS 424.77 | KX590781.1 |
| *Sporothrix euskadiensis* | VPRI43754 | MW075144.1 |
| *Sporothrix fraxini* | KFL21BS16bRJHV | MH283530.1 |
| *Sporothrix fumea* | CBS 129712 | KX590788.1 |
| *Sporothrix fusiformis* | KFL43916RJSR | MH283518.1 |
| *Sporothrix globosa* | CBS 120340 | KP101459.1 |
| *Sporothrix gossypina* | ATCC 18999 | KX590789.1 |
| *Sporothrix guttuliformis* | CBS 437.76 | KX590807.1 |
| *Sporothrix humicola* | CBS 118129 | KX590808.1 |
| *Sporothrix inflata* | 2 PB-2018 | MH283521.1 |
| *Sporothrix lignivora* | CBS 119147 | KP017107.1 |
| *Sporothrix luriei* | ATCC 18616 | AM747302.1 |
| *Sporothrix macroconidia* | CXY1894 | MH592598.1 |
| *Sporothrix mexicana* | Ss 132 | JF811340.1 |
| *Sporothrix narcissi* | CBS 138.50 | KX590791.1 |
| *Sporothrix nebularis* | CMW27319 | OK165572.1 |
| *Sporothrix nigrograna* | VPRI43755 | MW075145.1 |
| *Sporothrix nothofagi* | CMW 37658 | KX590810.1 |
| *Sporothrix pallida* | CBS 131.56 | KX590811.1 |
| *Sporothrix palmiculminata* | CBS 119590 | KX590794.1 |
| *Sporothrix phasma* | CBS 119721 | KX590795.1 |
| *Sporothrix polyporicola* | CBS 669.88 | KX590796.1 |
| *Sporothrix prolifera* | CBS 251.88 | KX590797.1 |
| *Sporothrix protearum* | CMW:50506 | MG431429.1 |
| *Sporothrix pseudoabietina* | CXY1937 | MH592601.1 |
| *Sporothrix rapaneae* | CMW40369 | KU639609.1 |
| *Sporothrix resoviensis* | KFL204ABRZN16AO | MH741228.1 |
| *Sporothrix roztoczensis* | KFL96So | OP589055.1 |
| *Sporothrix schenckii* | UTHSC 04-1064 | AM399014.1 |
| *Sporothrix silvicola* | KFL48So | OP589050.1 |
| *Sporothrix smangaliso* | CXY1937 | MF043586.1 |
| *Sporothrix splendens* | CMW:50507 | MG431431.1 |
| *Sporothrix stenoceras* | CBS 798.73 | KX590782.1 |
| *Sporothrix stylites* | CBS 118848 | KX590812.1 |
| *Sporothrix tumida* | KFL55RJ | OP589058.1 |
| *Sporothrix undulata* | KFL398DB16RJEG | MH741239.1 |
| *Sporothrix variecibatus* | CBS 121961 | KX590813.1 |
| *Sporothrix villosa* | SNM188 | MZ019543.1 |
| *Sporothrix zambiensis* | CMW:29078 | MG431432.1 |
